# Significant difference in gut microbiota *Bifidobacterium* species but not *Lactobacillus* species in colorectal cancer patients in comparison with healthy volunteers using quantitative real-time PCR

**DOI:** 10.1371/journal.pone.0294053

**Published:** 2024-11-27

**Authors:** Fahime Esfandiari, Bita Bakhshi, Tayebe Shahbazi, Elahe Derakhshan-nezhad, Mahboube Bahroudi, Sara Minaeeian, Mina Boustanshenas, Forough Alborzi, Behnam Behboudi, Mohamad Sadegh Fazeli

**Affiliations:** 1 Department of Bacteriology, Faculty of Medical Sciences, Tarbiat Modares University, Tehran, Iran; 2 Faculty of Medicine, Mashhad University of Medical Sciences, Mashhad, Iran; 3 Antimicrobial Resistance Research Center, Iran University of Medical Sciences, Tehran, Iran; 4 Division of Gastroenterology, Department of Surgery, Imam Khomeini Hospital Complex, Tehran University of Medical Sciences, Tehran, Iran; 5 Division of Colon and Rectal Surgery, Department of Surgery, Imam Khomeini Hospital Complex, Tehran University of Medical Sciences, Tehran, Iran; Wageningen Universiteit, NETHERLANDS, KINGDOM OF THE

## Abstract

**Background:**

Colorectal cancer (CRC), with a growing incidence trend, is one of the most diagnosed cancers and the second cause of cancer-related deaths worldwide. The literature has frequently focused attention on the correlation between the gut microbiota imbalance and CRC. The genera *Lactobacillus* and *Bifidobacterium* have recently received increasing attention because of their potential in restoring alterations in the gut microflora. Therefore, this study aimed to quantitatively evaluate the presence of lactobacilli and bifidobacterial strains in the fecal samples of CRC patients compared to healthy volunteers.

**Methods:**

From 2018 to 2019, 25 confirmed CRC patients and 25 age- and gender-matched control subjects were enrolled in the study. Bacterial DNA was extracted from the fecal samples and the presence of lactobacilli and bifidobacterial strains were quantitatively determined using quantitative real-time PCR using genus-specific 16S rDNA primers.

**Results:**

A significant decline in the abundance of bifidobacteria in CRC patients compared to healthy individuals (p value<0.003) was observed; however, no significant difference was observed between the two groups regarding the abundance of lactobacilli (p value<0.163). Correlation analysis showed a positive association between the lack of genetic history of CRC and the numbers of gut bifidobacteria and lactobacilli.

**Conclusion:**

As a putative gut probiotic, depletion of bifidobacteria showed significant correlation to the development and progression of CRC; therefore, therapeutic use of these probiotic bacteria could be considered a possible adjuvant approach in disease management through modulation of the microbiota.

## Introduction

Colorectal cancer (CRC) is one of the most common cancerous diseases worldwide. Globally, colorectal cancer is the third most common cancer diagnosed in both men and women [1, 2]. CRC is the third most common cause of cancer death in both men and women in the United States, and ranks second when men and women are combined. In 2020, approximately 147,950 CRC cases were diagnosed and 53,200 deaths were reported [3]. Despite the known benefits of screening and early diagnosis of CRC, the incidence of CRC has been steadily increasing in recent years, especially in developing countries [4]. Importantly, the global incidence of CRC is estimated to reach 2.2 million new cases by 2030. Thus, given its alarming prevalence and aggressive nature, CRC deserves international attention to further focus on understanding the disease and discover other treatment modalities to curb it [5].

CRC refers to tumors that start in the colon and develop altogether to the rectum. The risk of developing CRC gradually increases in the fourth decade of life and reaches its peak around the age of 70, thus, 90% of CRC cases occur after the age of 50 [6]. The majority of CRC cases are sporadic with no family history, however, some potential risk factors, like genetic and environmental factors, influence the risk of developing CRC [7]. Familial cancer syndromes account for approximately 6% of all CRC cases, induced by a known genetic mutation that could be inherited. Familial adenomatous polyposis (FAP) syndrome, which is caused by a mutation in the APC gene, is responsible for 1% of CRC [7, 8]. Lynch syndrome or hereditary nonpolyposis colon cancer (HNPCC), which is caused by mutations in one of four DNA mismatch repair genes (including MLH1, MSH2, MSH6, and PMS2), is responsible for 5% of colon cancers [9]. CRC is mainly developed on the left side of the colon in the sigmoid and rectum; however, research has shown a slow transmission of cancer to the right side of the colon. Synchronous tumors, which occur simultaneously and independently, develop in 5% of patients, while 3 to 5% of CRC patients suffer from metachronous tumors that develop as secondary tumors following the resection of the first one [10].

People with a family or personal history of some underlying diseases are usually at higher risk of developing CRC, including a family history of colorectal cancer in first-degree relatives, a personal history of adenomatous polyps, a history of IBD (inflammatory bowel disease), or a history of known familial cancer syndromes such as FAP or HNPCC [11]. Unhealthy dietary habits (including diets rich in processed and red meat, high-fat and low-fiber diets), sedentary lifestyles, excessive alcohol consumption, obesity, and excessive tobacco use are lifestyle factors associated with the risk of CRC in developing countries [12, 13]. It is worth noting that all these environmental factors have a direct impact on the intestinal microbiota composition [14]. Microbiota is a valuable, complex community of different bacterial populations with a mutualistic relationship, which reside in the intestinal epithelial barriers of humans and other animals and are essential for many physiological processes in the host [14–16]. Microbiota accomplishes different functions in the host at many levels, such as producing various important metabolites, protecting against pathogens invasion, and preventing the modulation of the local environment by controlling the overgrowth of some toxic bacterial groups [17]. In addition, a healthy human intestinal microbiota is essential for activating the host immune system, harvesting energy, and shaping the intestinal epithelium [1, 14]. Conversely, gut microbiota dysbiosis (imbalance in the gut microbiota) changes the host’s physiological functions, leading to a variety of diseases [18] ranging from intestinal disorders, like CRC, irritable bowel syndrome (IBS), and inflammatory bowel disease (IBD), to other systemic disorders such as obesity, malnutrition, diabetes, metabolic syndrome, and rheumatoid arthritis [19, 20]. Gut microbiota is predominantly composed of high levels of strictly anaerobic bacteria, including *Bacteroides*, *Eubacterium*, *Bifidobacterium*, *Fusobacterium*, *Peptostreptococcus*, and *Atopobium*, while a minor portion of its inhabitants are facultative anaerobes, such as *Enterobacteriaceae*, enterococci, lactobacilli, and streptococci [21].

Maintaining this structure is vital for intestinal homeostasis because the structural and metabolic functions of the gut microbiota prevent intestinal colonization by pathogens. Numerous emerging studies on the relationship between gut microbiota and CRC have described the critical role of intestinal microbiota in colorectal carcinogenesis [22]. These studies have reported enrichment or depletion of some microbial taxa in patients with CRC compared to healthy controls [1, 22]. Thus, microbiome alterations might be promising biomarkers for the early detection of CRC. On the other hand, previous research findings suggest that modulation of the gut microbiome could be a new strategy for the prevention and targeted therapy of colorectal cancer [1]. In recent years, much attention has been paid to understanding the composition of the intestinal microbiota of CRC patients to develop strategies aimed at manipulating microbiota for the benefit of host health and disease prevention and treatment [23].

Bifidobacterium species and Lactobacillus Species are the 2 most interested species among bacteria used as probiotics. Moreover, the 2 mentioned bacterial genus are suspected to have contribution in CRC prevention. Bifidobacterium bifidum has been reported to inhibits the development and progression of CRC in multiple ways, improvement of vitamin metabolism, and enhancing the efficacy of immune system [24], and also enhances the efficacy of antitumor immunotherapy by improving the local microenvironment [25]. Moreover, Bifidobacterium can promote the expression of the proapoptotic gene BAX in CRC, reduce the expression of Bcl-2, and promote apoptosis of cancer cells. Lactobacillus is also a probiotic strain known for its anti-inflammatory and anticancer characteristics [26]. Furthermore, some other studies have recently reported that this strain also displays antitumoral properties in a DMH-induced CRC model [26]. The current study aimed to enumerate their actual population in the bowel of CRC patients and healthy volunteers and highlight their probable correlation with CRC in Iranian population.

## Materials and methods

### Ethical approval

The project was approved by the Tarbiat Modares University Human Ethics Committee (IR.MODARES.REC.1398.011) and all methods were performed in accordance with the relevant guidelines and regulations. We received a written signed consent form from all study participants for using fecal samples and data obtained through the lifestyle, underlying diseases, and family history questionnaires as well as for publishing the analysis results.

### Study participants and sample collection

Sampling was performed for 8 months from 2018 to 2019. Stool samples were collected from confirmed CRC patients admitted to the surgical ward of Imam Khomeini hospital as well as from healthy volunteers who were gender and age-matched. In this study, the CRC cohort was composed of patients who presented with the main complaints of constipation (persistent, difficult, infrequent, or seemingly incomplete defecation), diarrhea (stool weight>200 g/d) [27], iron-deficiency anemia (Hb<12 in premenopausal women, Hb<13 in men, and ferritin<15) [28, 29], or rectal bleeding, and then their colorectal cancer was confirmed by colonoscopy, biopsy, and subsequent pathological examinations (positivity for CK20 and negativity for CK7). All pathological examinations were done by an expert pathologist. Patients diagnosed with colon or rectal adenocarcinoma underwent a staging process to determine the extent and location of cancer. Disease staging was assigned according to tumor-node-metastasis (TNM) staging system described by the American Joint Committee on Cancer (AJCC). Accordingly, Stage I disease includes adenocarcinomas that are invasive through the muscularis mucosa but are confined to the submucosa or the muscularis propria in the absence of nodal metastases. Stage II disease consists of tumors that invade through the bowel wall into the subserosa or non-peritonealized pericolic or perirectal tissues or into other organs or tissues or through the visceral peritoneum without nodal metastases. Stage III disease includes any T stage with nodal metastases, and stage IV disease denotes distant metastases [30]. The staging process for both colon and rectal cancers involved computed tomography (CT) scans of the chest, abdomen, and pelvis to reject the possibility of distant metastatic disease. Blood staging involved testing for carcinoembryonic antigen (CEA). It should be noted that CEA is not specific for CRC, but could be used in the follow-up of patients undergoing tumor resection to diagnose recurrence. The staging process of rectal cancer also involved an additional examination, magnetic resonance imaging (MRI) or ultrasonography, to evaluate the extent and degree of tumor invasion in the bowel wall as well as the involvement of lymph nodes [31].

In this study, participants also completed questionnaires that examined their lifestyle as well as the underlying diseases of volunteer patients. Exclusion criteria were as follows: consumption of probiotic products during the last 20 days before sample collection [32]; use of antibiotics during the last 45 days [33]; and use of anti-inflammatory and steroid drugs during the last 45 days [32]. The collection of stool samples was done before any chemotherapy or radiography.

The healthy volunteers were selected so that they pair-wise matched the patient group according to obesity, sex, age and etc. Fecal samples were also taken from healthy volunteers as control subjects at the same time points. All fecal specimens were collected in sterile cups instantly after defecation and delivered to the laboratory within 2 hours after collection. Upon arrival at the microbiology laboratory, fecal samples were immediately transferred to a freezer and kept at −80 °C.

### Fecal samples processing and total DNA extraction

To extract bacterial DNA, 500 mg of each fecal sample was measured by a digital scale and prepared for the next step. Total genomic DNA was efficiently extracted from all stool specimens using Favor Gen DNA Stool kit (Stool DNA Isolation mini Kit) according to the manufacturer´s protocol. DNAs quality and concentration were determined by a Nanodrop spectrophotometer (Nanodrop Technologies, Wilmington, DE, USA) and agarose gel electrophoresis. All extracted DNAs were immediately stored at -20 °C.

### Standard bacterial strains

Reference strains used in this study were obtained from the American Type Culture Collection (*L*. *acidophilus* ATCC 4356, *B*. *bifidum* ATCC 29521).

### Primers, real-time qPCR, and microbial quantification

Previously published primers for detection of *Lactobacillus* and *Bifidobacterium* spp. at the genus-level were used to amplify 204 and 85 bp amplicons of 16S rRNA genes from each genus, respectively (Table 1) [15]. Selected primers were re-checked by SILVA High Quality Ribosomal RNA database (https://www.arb-silva.de) and Primer BLAST software (https://www.ncbi.nlm.nih.gov/tools/primer-blast) for inclusivity, specificity and probable mismatches. Primers were further analyzed by oligo analyzer tool (https://www.idtdna.com/pages/tools/oligoanalyzer) for melting temperature and secondary structures. The annealing temperature was considered 5 to 8 °C higher than melting temperature to overcome the non-specific off-target amplifications.

**Table 1:** 16S rRNA primers used to analyze the abundance of *Lactobacillus* and *Bifidobacterium* spp in fecal samples.

Target Bacteria	Primer	Oligonucleotide Sequence (5^′^-3^′^)	Size (bp)	Product Size (bp)	Reference
*Lactobacillus* spp.	Primer F	GTCTGATGTGAAAGCCYTCG	20	204	[13]
	Primer R	CCAGGGTATCTAATCCTGTTYG	22		
*Bifidobacterium* spp.	Primer F	GGTTAACTCGGAGGAAGG	18	85	[13]
	Primer R	GTACCGGCCATTGTAGCA	18		

Real-time PCR was performed using Brilliant SYBR Green QPCR Master Mix to measure the quantity of the two bacterial groups in Stargen Mx3000 real-time PCR cycler (Qiagen Corbett, Hilden, Germany) using genus-specific 16S rDNA primers. All qPCR experiments were performed in three independent tests, and the mean values of triplicate experiments were used for calculations and analysis. PCR reaction mixtures with a total volume of 20 μL contained 2 μL (50ng/μL) of template DNA, 10 μL of qPCR Master Mix (Takara Bio, Shiga, Japan), 1 μL of forward primer, 1 μL of reverse primer, and 6 μL of sterilized ultra-pure water. The concentration of DNA extracted from stool samples, were assessed with nanodrop instrument and identical amounts of DNA (100ng) was used in each real-time PCR reaction to overcome DNA yield and concentration differences. Real-time qPCR was performed under the following thermal cycling conditions: an initial denaturation step at 95 °C for 30 s, followed by 40 cycles of denaturation at 95 °C for 10 min, annealing at 60°C for 40 s, and extension at 70 °C for 30s. Negative controls containing all the reaction components except the template DNA were performed with each amplification reaction, and no detectable amplified DNA was found. Finally, the amplification curve and melting temperature of the samples were recorded. The presented data were the mean values of triplicate real-time qPCR analysis.

**Fig 1:**
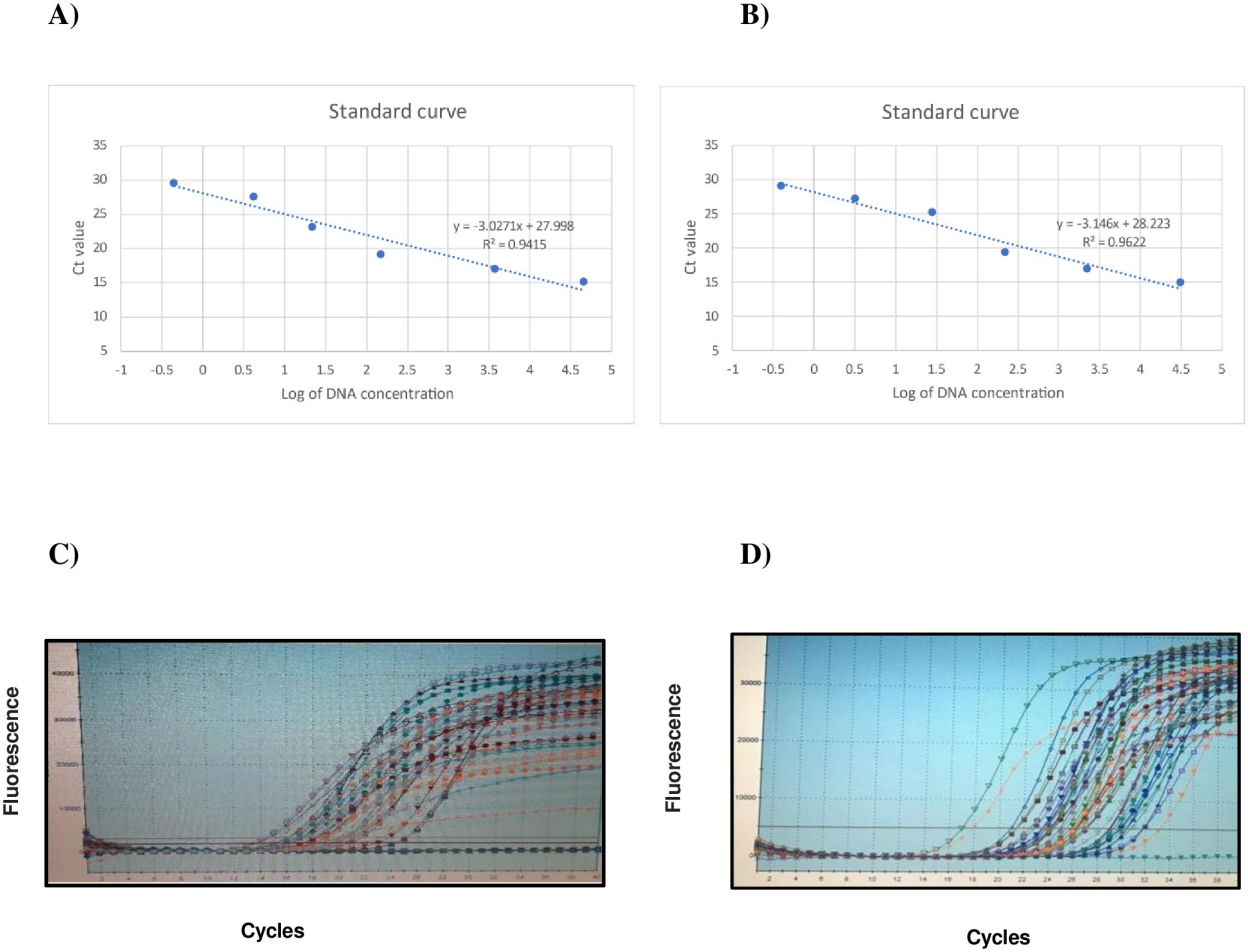
Standard curves constructed based on ten-fold serial dilutions of genomic DNA of A) Bifidobacterium, B) Lactobacillus. The CT-values corresponding to each DNA concentration were used to construct a standard curve. Sample amplification plot generated by Real-time qPCR of DNA extracted from stool of patients and healthy volunteers, C) Bifidobacterium, D) Lactobacillus.

To construct a standard curve, 10-fold serial dilutions were prepared from genomic DNA of known concentration obtained from pure culture of reference bacterial strains, and subjected to real-time PCR analysis. The CT-values corresponding to each DNA concentration were used to construct a standard curve (Table 2, Fig 1). Standard curves were created according to tutorial guides for performing relative quantitation of nucleic acid using real-time PCR technologies developed by Applied Biosystems (http://www.appliedbiosystems.com). Threshold cycle values (CT) obtained by real-time PCR were used to calculate the bacterial DNA concentration in each sample based on the standard curves which then normalized to the copy number of the 16S rRNA gene for each bacterial genus [34].

**Table 2:** Bacterial DNA used for construction of standard curve.

Lactobacillus pure culture (0.5 McFarland)		Bifidobacterium pure culture (0.5 McFarland)	
(Concentration of extracted DNA = 4.5×10^4^ pg/μl)		(Concentration of extracted DNA = 4.67×10^4^ pg/μl)	
Dilution	Ct value (mean)	Dilution	Ct value (mean)
1	14.91	1	15.1
1:10	16.84	1:10	17.02
1:100	19.31	1:100	19.1
1:1000	25.12	1:1000	23.13
1:10000	27.1	1:10000	27.6
1:100000	29.03	1:100000	29.53

### Converting DNA concentration to bacterial copy number

The bacterial DNA concentration in each sample was converted to bacterial copy number according to formula:

$$\text{number}\;\text{of}\;\text{copies}\;(\text{molecules})=\frac{\text{X}\;\text{ng}*\;6.0221\times 10^{23}\text{molecules}/\text{mole}}{{\left(\text{N}\;*\;\text{660}\;\text{g/mole}\right)}^{†}*1\times 10^{9}\text{ng/g}}$$

Where X is the amount of DNA (ng), N is the length of dsDNA Bifidobacterium/Lactobacillus genome, 660 g/mol is the average mass of 1 bp dsDNA, 6.022 x 10^23^ is Avogadro’s constant and 1 x 10^9^ is the conversion factor [35].

### Statistical analysis

Statistical analysis was carried out using SPSS software, Version 22.0 (SPSS Inc., Chicago, IL, USA). Considering the limitations of number of patients with the required criteria, the sample size was calculated and its robustness was assured according to the book by Tabachnick B.G. and colleagues (2012) [36].

In statistical analysis, among all the variables included in the lifestyle questionnaire, the age variable was quantitative, and the rest were qualitative. Kolmogorov-Smirnov (K-S) test was used to test the normality of the distribution of the variables. Student’s *t*-test was used to compare to the age between the two groups of CRC patients and healthy volunteers. The Chi-square test and Fisher’s exact test were used to test the similarity in the levels of variables between the two groups, including sex, alcohol consumption, and tobacco use. These tests and Binomial test were also used to evaluate the underlying diseases questionnaire that assessed the genetic/personal history of colorectal cancer, familial polyposis cancer, hereditary nonpolyposis cancer, and adenomatous polyps in patients and their family or first-degree relatives, meat, fast food, and vegetables.

Mann-Whitney test was used to assess the correlation between the number of bacteria and food consumption style in both patient and healthy groups.

In order to increase the accuracy, we also performed this comparison in R software and used the PERMANOVA package, and the p value was <0.05. As a result, there is a significant difference between the two groups in terms of the number of bacteria.

## Results

### Patients and staging process of CRC

The study population consisted of 20 patients with CRC and 20 healthy volunteers. To prevent possible errors, the total number of volunteers participating in this study reached 50 people, including 25 CRC patients and 25 healthy people. Five cases out of 25 patients were in stage 4 disease, and 20 cases were in stage 3 disease.

### Inter-group qPCR analysis of *Lactobacillus* and *Bifidobacterium* between CRC and healthy cohorts

**Fig 2:**
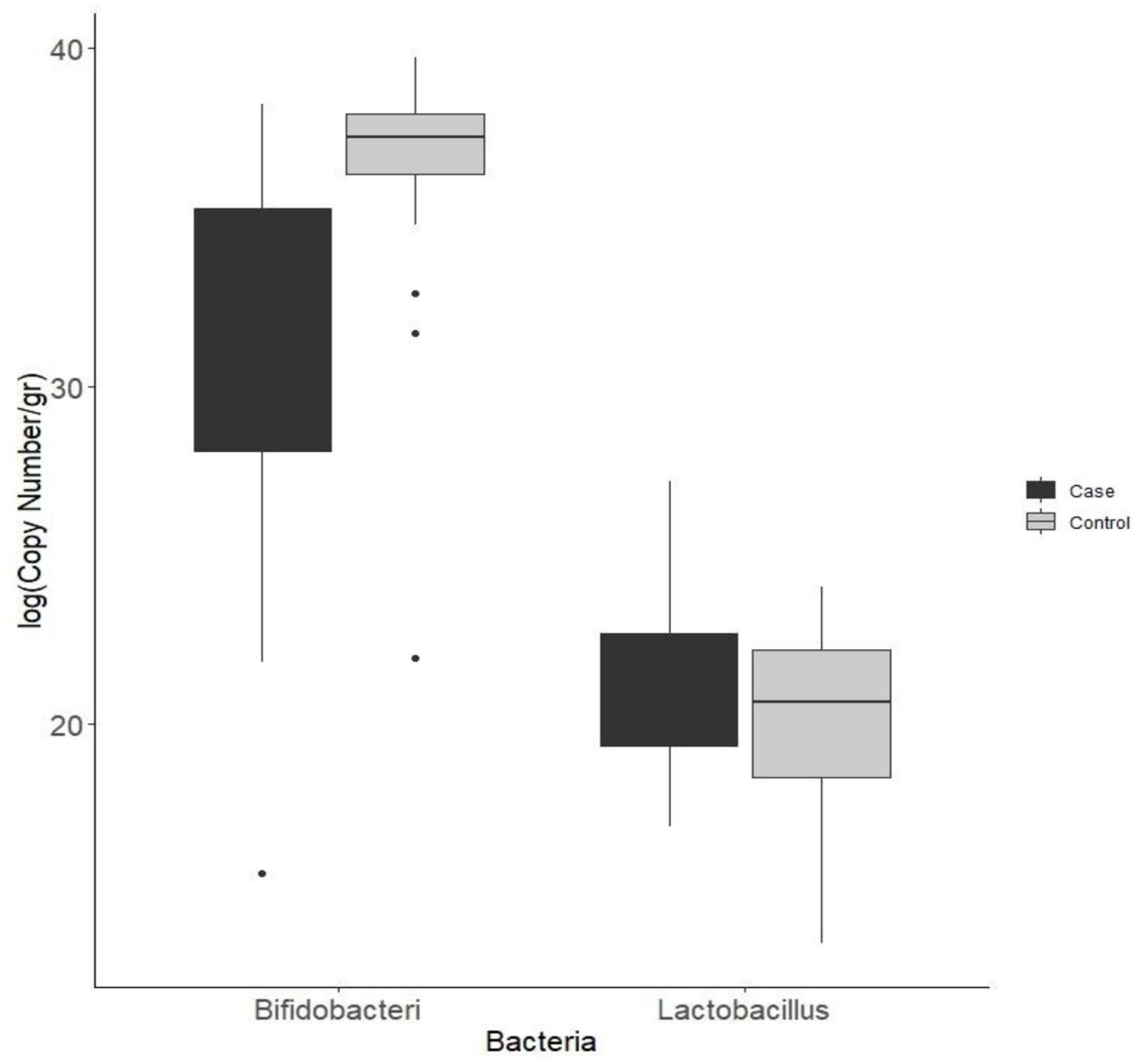
*Lactobacillus* and *Bifidobacterium* quantified by real time qPCR and expressed as log (copy number) of bacterial groups per gr stool in patient and healthy volunteers.

In this case-control study, quantitative PCR (qPCR) analysis was performed to evaluate the differences in fecal microbiota composition between CRC patients and healthy individuals in terms of the frequency of *Lactobacillus* and *Bifidobacterium* bacteria (Fig 1). After removing outliers, the inter-group analysis indicated a meaningful difference in the abundance of *Bifidobacterium* bacteria between the two groups of CRC patients and healthy individuals and the number of *Bifidobacterium* strains was significantly higher in healthy individuals compared with CRC patients (*p* value<0.003). But no significant difference was observed between the two groups regarding the mean frequency of *Lactobacillus* bacteria (*p* value<0.163) (Fig 2, Table 3).

**Table 3:** Statistical results of the CRC patients and healthy individuals.

Bacterial Species	Mean ± standard deviation		Leven’s test		*t*-test for Equality of Means		
	Case (N = 25)	Control (N = 25)	F	sig		T	Sig
*Lactobacillus*	4.49 × 10^9^ ± 1.41 × 10^6^	4.60 × 10^9^± 7/52 × 10^4^	7.324	0.009	Equal variances assumed	1.41	0.163
*Bifidobacterim*	3.336 × 10^15^ ± 9.31× 10^10^	3.55× 10^16^ ± 5.00 × 10^11^	14.449	0.0001	Equal variances assumed	-3.159	0.004

### Intra-group qPCR analysis of *Lactobacillus* and *Bifidobacterium* in CRC patients and healthy volunteers

Intra-group analysis of the frequency of *Lactobacillus* and *Bifidobacterium* bacteria between members of each group (CRC patients and healthy individuals) indicated significant differences in the abundance of both bacterial genera between CRC patients (*p*-value<0.001); however, no significant difference was observed regarding the abundance of *Lactobacillus* and *Bifidobacterium* genera between healthy volunteers (*p*-value > 0.05).

### Lifestyle questionnaire (LSQ) analysis

Analysis of participants’ lifestyle data collected through LSQ showed no significant difference between the two groups of CRC patients and healthy individuals regarding consumption patterns of alcohol, meat, fast food, vegetable, and legume (*p*-value > 0.05). Also, there was no significant difference between the two groups in terms of the smoking pattern (*p*-value > 0.05) (Table 4).

**Table 4:** Summary of the results that examined the relationship between lifestyle data and colorectal cancer.

Variable	Test	*P-*Value
Age	*t*-Test	0.357
Gender	Chi-square test	0.89
Alcohol consumption	Fisher’s exact test	0.463
Smoking	Chi-square test	0.25
Meat consumption	Fisher’s exact test	0.143
Fast-food consumption	Fisher’s exact test	0.690
Vegetable consumption	Fisher’s exact test	0.901

Mann-Whitney test was used to assess the correlation between the number of bacteria and food consumption style in both patient and healthy groups. Accordingly, there was no significant correlation between different foods consumption style and the number of Bifidobacterium and Lactobacillus strains in the stool of CRC patients and healthy group (p-value > 0.05) (Table 5).

**Table 5:** Statistical correlation between food consumption style and the number of *Bifidobacterium* and *Lactobacillus* in both patient and healthy groups.

Food consumption style	Patient group (*P-*Value)		Healthy group (*P-*Value)	
	*Bifidobacterium*	*Lactobacillus*	*Bifidobacterium*	*Lactobacillus*
**Meat Consumption**	0.840	0.190	0.720	0.500
**Fast Food Consumption**	0.495	0.423	0.27	0.198
**Vegetables Consumption**	0.225	0.225	0.936	0.058
**Legume Consumption**	0.058	0.497	0.695	0.748

### Analysis of data regarding underlying diseases in CRC patients

The related questionnaire was designed to study the genetic background of underlying diseases in CRC patients. In this study, only of CRC volunteers were involved; thus, the study was limited to the frequency and percentage of diseases among CRC volunteers. Analysis of data regarding underlying diseases in CRC patients showed significant difference regarding family history of polyposis cancers (*p*-value < 0.05). There was no significant difference in terms of family history of colorectal cancer /non-polyposis cancers/adenomatous polyps (*p*-value > 0.05) (Table 6).

**Table 6:** Statistical analysis of the underlying disease in CRC patients.

Underlying Disease	Number of Patients with Underlying Disease	Number of Patients without Underlying Disease	*P-*Value
Family history of colorectal cancer	8	17	0.108
Crohn’s disease	1	24	LN*
Family history of polyposis cancers	6	19	0.015
Family history of non-polyposis cancers	16	9	0.230
Family history of Adenomatous polyps	3	22	0.210
Adenomatous polyps	2	23	LN*

LN*: Low Number, No statistical analysis due to low number of cases with underlying disease

### Correlation between underlying diseases in CRC patients and bacterial estimates by qPCR

Correlation analysis showed a positive association between the lack of genetic history of colorectal cancer and the presence of high numbers of *Lactobacillus* (*p*-value = 0.001) and *Bifidobacterium* (*p*-value = 0.000) strains in the stool of CRC patients. In other words, the frequency of both bacterial genera in the stool of CRC volunteers without a genetic history of colorectal cancer was higher compared to patients with a genetic history of the disease.

The effect of the disease stage on the number of *Lactobacillus* and *Bifidobacterium* bacteria was also evaluated, and the results indicated a significant correlation between the numbers of both bacterial genera in the stool of CRC volunteers and the disease stage. In fact, patients in the third stage of the disease harbored higher numbers of *Lactobacillus* and *Bifidobacterium* strains in their stool compared to patients in the fourth stage of the disease.

Correlation analysis also indicated a significant association between the family history of polyposis cancers/adenomatous polyps and the lower number of fecal *Lactobacillus* strains (*p*-value < 0.05), while no significant difference was observed in the amount of *Bifidobacterium* strains (*p*-value > 0.05). There was no significant correlation between the family history of non-polyposis cancers and the number of *Lactobacillus* and *Bifidobacterium* strains in the stool of CRC patients (p-value > 0.05) (Table 7).

**Table 7:** Statistical analysis of the underlying disease questionnaire of CRC patients in terms of *Bifidobacterium* and *Lactobacillus*.

Underlying Disease	No. of Patients with Underlying Disease	No. of Patients without Underlying Disease	*P-*Value (in terms of *Bifidobacterium*)	*P-*Value (in terms of *Lactobacillus*)
Family history of Colorectal cancer	8	17	0.000	0.001
Crohn’s disease	1	24	0.332	LN*
Family history of Polyposis cancers	6	19	0.065	0.000
Family history of non-Polyposis cancers	16	9	0.174	0.113
Family history of Adenomatous polyps	3	22	0.210	0.008
Adenomatous polyps	2	23	0.271	LN*

LN*: Low Number, No statistical analysis due to low number of cases with underlying disease

## Discussion

Many previous studies have indicated that substantial changes in the abundance of specific genera and species in the intestinal microbiota of patients or animal models lead to many chronic diseases such as obesity [37–40], diabetes [41–43], IBD (inflammatory bowel disease) [44, 45], autism [46–48], and even cancer [49–51]. In addition, growing evidence has demonstrated that dysbiosis of intestinal microbiota plays a critical role in CRC initiation, progression, and metastasis [52, 53]. Additionally, less bacterial diversity and richness have been reported in the stool of CRC patients compared to the stool of healthy individuals [14, 54, 55]. Furthermore, numerous studies on human microbiota have shown that intestinal microbiotas of CRC patients are structurally different in both fecal and mucosal levels compared with matched microbiotas of healthy individuals [14, 56]. Studies have shown that strategies such as fecal microbiome transplantation (FMT), administration of probiotics, and therapeutic diets for some gastrointestinal disorders, including inflammatory bowel disease, metabolic diseases, and even cancer, are approved treatments for the treatment of such diseases [57].

At present, both *Bifidobacterium* and *Lactobacillus*, as common human intestinal microflora, are the most commercially available genera of probiotic bacteria. Considering the significance and prevalence of colorectal cancer worldwide and the beneficial effects of probiotics in preventing the progression and improving CRC, this study aimed to investigate the differences in the number of *Lactobacillus* and *Bifidobacterium* bacteria in the stool of patients with colorectal cancer and healthy volunteers.

In this study, no statistically significant difference was detected regarding the number of fecal lactobacilli between CRC patients and healthy individuals, but a remarkable difference was observed in the number of intestinal bifidobacteria between CRC patients and healthy volunteers. The number of bifidobacteria was significantly higher (about 10 folds) in healthy individuals compared with CRC patients.

In contrast to this study’s results, O’Keefe et al. (2007) compared stool samples of people with colorectal cancer and healthy people and found that the frequency of *Bifidobacterium* spp. was significantly higher in CRC patients [58]. In addition, Scanlan et al. (2009) found that the frequency of lactobacilli was higher in people at lower risk of developing colorectal cancer compared to those at higher risk of CRC [59]. Moreover, Sobhani et al. (2011) found that there was no significant difference in the amount of dominant and subdominant species, including *Lactobacillus* and *Bifidobacterium*, between people with colorectal cancer and normal individuals [49]. It can be concluded that controversial results may be due to different risk factors of colorectal cancer around the world depending on the geographical variations and biological features of predisposing genetic factors as reported by Ulanja et al. [60]. On the other hands, population differences in gene expression could contribute to some of the observed differences in susceptibility of individuals to common diseases and response to drug treatments [61, 62]. Conducting similar studies in different populations is mandatory in search for the involvement of composition of microbiota including the Bifidobacteria and Lactobacilli, and different genetic backgrounds in CRC development and progression.

To our knowledge, no previous report has been published on probable modulatory role of dominant microflora in CRC development in Iran. These data together with previously published and upcoming studies would help fulfill some of the missing parts of the triggering factors of CRC puzzle in different populations.

Similar to this study, Sobhani et al. (2011) indicated significant differences regarding the number and type of dominant fecal microbial species between patients with CRC-associated dysbiosis, depending on the disease severity and tumor stage [49]. Moreover, Schulz et al. (2014) investigated the role of dysbiosis in CRC development in mice through antibiotic experiments. They found that changes in intestinal microbiota during dysbiosis were associated with tumorigenesis and appeared to be strongly involved in this process [51].

According to Commane et al. (2005), knowledge about the anti-cancer properties of probiotics supports the hypothesis that probiotics inhibit cancer in its early stages. Their study results demonstrated that the use of probiotics affected cancer in the early stages by reducing the epithelial pressure on active carcinogens [63].

Using colon carcinogen models such as 1,2-dimethylhydrazine (DMH) in the rat colon, Kumar et al. (2010) reported that oral probiotics could exhibit anti-carcinogenic properties in the intestinal tract by counteracting the toxicity of substances acting through DNA damage [64].

According to a study by Rafter et al. (2007), *Lactobacillus* and *Bifidobacterium* may contribute to modulating intestinal metabolism and exhibit a protective effect against CRC, although their study results showed that these effects were significantly stronger in animals than in humans [65].

Lifestyle, such as dietary habits, is one of the remarkable factors that influence the amount and diversity of intestinal microbiota and also the development or progression of colorectal cancer [22]. In addition, underlying diseases and family history of some diseases also affect the development or progression of CRC [66–68]. Analysis of participants’ lifestyle data collected through LSQ indicated no significant difference between the two groups in terms of consumption of red meat, alcohol, fast food, vegetables, legumes, and smoking.

Even though red meat has been identified as a cancer-promoting factor, the results of research studies do not always literally support the relationship between the consumption of red or processed meat and the risk of gastrointestinal cancers [69, 70]. In a case-control prospective study conducted by Ananthakrishnan et al. (2015), a pooled analysis of CRC cases and controls indicated no significant relationship between red meat consumption and colorectal cancer [71]. In contrast, a strong positive relationship was found by Zhao et al. (2017) in a meta-analysis of studies on the association between red meat consumption and gastric cancer [72].

In contrast to the present study results, some case-control and prospective cohort studies, but not all, have shown statistically significant associations between excessive alcohol consumption and an increased risk of CRC, while several studies have indicated either inconsistent or just weak associations between the two [73]. Smyth et al. (2015) in a prospective cohort study in 12 countries with various economic levels revealed that regular drinking was related to an increased risk of alcohol-induced cancers such as CRC [74]. Bagnardi et al. (2015) in a dose-response meta-analysis study investigated the effect of alcohol consumption on 23 cancer types including CRC. They reported the relative risks of developing CRC for heavy drinkers compared with non-drinkers and occasional drinkers [75].

Analysis of underlying diseases and family history of some diseases of CRC patients indicated a significant association between a family history of polyposis cancers and adenomatous polyps with reduced number of fecal *Lactobacillus* strains, but no significant difference was observed in terms of *Bifidobacterium* strains.

Liang et al. (2009) reported that Crohn’s disease in patients was associated with a decrease in the population of bifidobacteria and butyrate-producing bacteria [76]. The same researchers found a decrease in the frequency of *Firmicutes* and *Actinobacteria* belonging to the order *Bifidobacteriales* in patients with adenomatous polyps [76]. Mangifesta et al. (2018) observed no significant difference in bacterial populations between healthy mucosa and polyp-associated mucosa, although they observed a moderate reduction in the abundance of *Actinobacteria* and a concomitant moderate increase in the amount of *Firmicutes* in patients compared to healthy subjects [77]. These together signify the probable contribution of other variables, such as diet, host genetic determinants, and ethnic background, on the fecal microbiome composition and subsequently the CRC.

## Conclusion

In summary, from the results obtained by our research on the abundance of *Bifidobacterium* and *Lactobacillus* strains concerning CRC, it was found that the number of bifidobacteria was significantly higher in healthy individuals compared with CRC patients. On the contrary, no significant difference was observed between the two groups regarding the abundance of Lactobacilli. We hypothesize that depletion of gut bifidobacteria can contribute to CRC development and risk. Accordingly, given the association of bifidobacterial depletion to CRC, it may provide a contributory biomarker to the disease status in the future. In addition, performing further studies, either in clinical trials or studies in animal models, are recommended to evaluate the precise role of regular ingestion of bifidobacterial probiotics to reduce or prevent CRC risk and/or complications.

### Limitation of study

The selection of patients was the most challenging step in this study. The patients were being selected according to definition and diagnostic criteria of CRC patients, but considering the exclusion criteria, majority of confirmed CRC cases were being excluded from the study due to consumption of probiotic products in 20 days or use of antibiotics/anti-inflammatory/steroid drugs in 45 days prior to sampling.
